# Supplementary material for: Intergenerational Perioperative Neurocognitive Disorder
Source: Biology (Basel). 2023 Apr 7;12(4):567. doi: 10.3390/biology12040567 (PMC10135810; doi:10.3390/biology12040567)
Supplement: Supplementary file 1 [file biology-12-00567-s001.zip › biology-2283938-supplementary.pdf]

Methods

Animals and Groups

All experimental procedures were approved by the University of Florida Institutional Animal Care and Use Committee. The study was conducted and data is reported in accordance with the ARRIVE guidelines [1]. All rats were housed under controlled illumination (12-h light/12-h dark, lights on at 7:00 AM) and temperature (23–24 °C) with free access to food and water.

Acute stress-like effects of sevoflurane (SEVO) in young adult male rats: Top panel of Figure 1 shows an overview of the study design. Twenty male Sprague–Dawley rats were purchased from Charles River (Wilmington, MA) and were randomized into four treatment groups (n = 5/group) using a randomization plan with a web-based generator, and the investigators were blinded to group assignments. The P60 male rats were kept in a temperature-controlled chamber to maintain body temperature at ~+37 ºC. The chamber was continuously supplied with 30% O_2 in air (1.5 L/min) during anesthesia. Anesthesia with sevoflurane was induced with 6% sevoflurane for 3 min followed by 2.1% sevoflurane for 177 min. A rectal temperature probe was placed in some animals to monitor body temperature. Gas monitoring was performed using a calibrated Datex side stream analyzer (Datex-Ohmeda, Helsinki, Finland), which samples from the animal chamber interior. Vehicle [intraperitoneal injection (I.P.)], Na^+-K^+-Cl^- (NKCC1) Cl^- importer inhibitor bumetanide (1.84 mg/kg, I.P.) [2,3], or K^+-2Cl^- (KCC2) Cl^- exporter

enhancer CLP290 [100 mg/kg, Per os (P.O.)] [4-6] were administered 30 min prior to the onset of anesthesia with SEVO (the Vehicle + SEVO, Bumetanide + SEVO and CLP290 + SEVO groups, respectively). Rats in the Vehicle + Control group were placed in a new cage and housed one per cage for an equivalent amount of time on P60. All rats were sacrificed 1 h after recovery from SEVO anesthesia on P60 or at an equivalent timepoint in the Vehicle + Control group to collect tissue samples.

Intergenerational effects of neonatal exposure to SEVO: Top panel of Figure 2 shows an overview of the study design. Adult male and female Sprague-Dawley rats purchased from Charles River (Wilmington, MA) were used as breeders. Within 24 h of delivery, litters were culled to 12 pups. At the age of 21 days, pups were weaned and housed in sex-matched pairs for the rest of the study.

The P5 male rats (generation F0) were kept in a temperature-controlled chamber to maintain body temperature at ~+37 ºC. The chamber was continuously supplied with 30% O_2 in air (1.5 L/min) during anesthesia. Anesthesia with SEVO was induced with 6% SEVO for 3 min followed by 2.1% sevoflurane for 297 min. This level of anesthesia was sufficient to induce loss of the righting reflex, but not sufficient to induce loss of the withdrawal reflex to a noxious stimulus. A rectal temperature probe was placed in some animals to monitor body temperature. Gas monitoring was performed using a calibrated Datex side stream analyzer (Datex-Ohmeda, Helsinki, Finland), which

samples from the animal chamber interior. Previously, we have shown that blood glucose and blood gas levels after 2.1% SEVO for 6 h were in the normal range.[3] The vehicle (the SEVO group) or bumetanide (1.84 mg/kg, I.P.; the Bumetanide + SEVO group) was given to animals 30 min prior to the onset of anesthesia with SEVO. Rats in the F0 control group were subjected to animal facility rearing only (the control group). They remained with their dams. The primary argument against the use of a control group for maternal separation during SEVO anesthesia was that anesthetized pups do not experience separation stress, while 5 h of maternal separation is a stressor. All animals except those in the control group received a subcutaneous injection of saline (1 mL/100 g) at 2.5 h of anesthesia with SEVO to prevent dehydration. On P90, the F0 male rats (8 rats/group) were mated with control female rats (8 female rats/group) to generate offspring (generation F1) of: 1) control males/control females (Ctrl*Ctrl); 2) SEVO males/control females (SEVO*Ctrl); and 3) Bumetanide + SEVO males/control females (BS*Ctrl). The female rats were kept alone throughout the entire gestation and postpartum rearing periods. A given P0 F1 offspring experimental group (n = 5/group/sex) included one to two rats produced by a given F0 breeding pair. Brain tissue samples were isolated from P0 F1 rats for gene expression analyses.

Tissue Collection

Rats were anesthetized with SEVO and decapitated. The trunk blood samples were collected and centrifuged at 4 °C, 1000 g for 15 min, and serum corticosterone was

measured using commercial ELISA kits (Cayman Chemical Company, Ann Arbor, MI) according to the manufacturer's instructions. [7-9] The hypothalamus was isolated by making an anterior cut at the level of the optic chiasm, a posterior coronal section anterior to the mammillary bodies, two sagittal cuts parallel to the lateral ventricles, and a dorsal horizontal cut at the level of the anterior commissure. The hippocampus was isolated from the respective blocks.[7] All tissue samples were placed in vials filled with *RNAlater* solution (Invitrogen, Carlsbad, CA, USA).

Measurement of Messenger RNA Levels

The messenger RNA (mRNA) levels in the hypothalamus and hippocampus were analyzed via reverse transcription-polymerase chain reaction (RT-PCR) in a StepOnePlus™ Real-Time PCR System (Applied Biosystems, Foster City, CA, USA), as previously described. [7-12] RNA was extracted from the samples using an RNeasy Plus Kit (Qiagen, Valencia, CA, USA), reverse transcribed with a high-capacity cDNA reverse transcription kit (Bio-Rad Laboratories, Hercules, CA, USA), and then analyzed via RT-PCR. Taqman probes specific for *Nkcc1*, *Kcc2*, corticotropin-releasing hormone (*Crh*), glucocorticoid receptor (*Gr*), mineralocorticoid receptor (*Mr*), *aromatase*, estrogen receptor α (*Erα*), estrogen receptors β (*Erβ*), DNA methyltransferase 3a (*Dnmt3a*), *Dnmt3b*, and *Dnmt1* mRNA were obtained from Applied Biosystems: *Nkcc1* (Rn00582505_m1), *Kcc2* (Rn00592624_m1), *Crh* (Rn01462137_m1), *Gr* (Rn00561369_m1),

Mr (Rn00565562_m1), *aromatase* (Rn00567222_m1), *Erα* (Rn01430446_m1), *Erβ* (Rn00562610_m1), *Dnmt3a* (Rn01027162), *Dnmt3b* (Rn01536414_g1), and *Dnmt1* (Rn00709664_m1). Data were normalized to glyceraldehyde-3-phosphate dehydrogenase (*Gapdh*) mRNA (Rn01775763_g1). Gene expression was calculated using the ΔΔCT method and data are presented as relative fold change from that of control animals.

Statistical Analyses

Statistical analyses were conducted on raw data using SigmaPlot 14.0 software (Systat Software Inc., San Jose, CA), which automatically checks if the data set meets test criteria (Shapiro-Wilk for normality test and Brown-Forsythe for equal variance test). Values are reported as mean ± SEM. Sample size calculations were done, assuming a range of anticipated differences in mean outcomes and standard error based on background data and past experience with similar measurements in Sprague-Dawley rats.[7-12] This analysis indicated that sample sizes of n = 5 rats/group for measurements in tissue samples were sufficient to detect differences between treatment groups, with effect sizes of ≥0.8, assuming an α level of 0.05. This translated to a mean difference of 10% in the serum levels of corticosterone and mRNA levels. Boxplots were used to identify outliers. No outliers were detected that were not in the plausible range of values for the outcomes; therefore, all data were maintained in analyses. We used one-way ANOVA to assess the differences in the mRNA and corticosterone levels. All

multiple pairwise comparisons were done using the Fisher Least Significant Difference method. $P < 0.05$ was considered significant.

References

1. Evered L, Silbert B, Knopman DS, Scott DA, DeKosky ST, Rasmussen LS; et al.: Nomenclature Consensus Working Group: Recommendations for the nomenclature of cognitive change associated with anaesthesia and surgery-2018. J Alzheimers Dis 2018; 66:1–10.
2. Kahle, K.T.; Staley, K.J. The bumetanide-sensitive Na-K-2Cl cotransporter NKCC1 as a potential target of a novel mechanism-based treatment strategy for neonatal seizures. *Neurosurg. Focus* **2008**, *25*, E22, https://doi.org/10.3171/foc/2008/25/9/e22.
3. Edwards, D.; Shah, H.P.; Cao, W.; Gravenstein, N.; Seubert, C.N.; Martynyuk, A.E. Bumetanide Alleviates Epileptogenic and Neurotoxic Effects of Sevoflurane in Neonatal Rat Brain. Anesthesiology **2010**, *112*, 567–575. https://doi.org/10.1097/aln.0b013e3181cf9138.
4. Gagnon, M.; Bergeron, M.J.; Lavertu, G.; Castonguay, A.; Tripathy, S.; Bonin, R.P.; Perez-Sanchez, J.; Boudreau, D.; Wang, B.; Dumas, L.; et al. Chloride extrusion enhancers as novel therapeutics for neurological diseases. Nat. Med. **2013**, *19*, 1524–1528. https://doi.org/10.1038/nm.3356.
5. Tang, B.L. The Expanding Therapeutic Potential of Neuronal KCC2. Cells **2020**, *9*, 240. https://doi.org/10.3390/cells9010240.
6. Lizhnyak, P.N.; Muldoon, P.P.; Pilaka, P.P.; Povlishock, J.T.; Ottens, A.K. Traumatic Brain Injury Temporal Proteome Guides KCC2-Targeted Therapy. *J. Neurotrauma* **2019**, *36*, 3092–3102. https://doi.org/10.1089/neu.2019.6415.
7. Ju, L.-S.; Yang, J.-J.; Morey, T.; Gravenstein, N.; Seubert, C.; Resnick, J.; Zhang, J.-Q.; Martynyuk, A. Role of epigenetic mechanisms in transmitting the effects of neonatal sevoflurane exposure to the next generation of male, but not female, rats. *Br. J. Anaesth.* **2018**, *121*, 406–416. https://doi.org/10.1016/j.bja.2018.04.034.
8. Ju LS, Yang JJ, Xu, N.; et al. Intergenerational effects of sevoflurane in young adult rats. Anesthesiology. 2019;131:1092–1109.
9. Ju, L.-S.; Yang, J.-J.; Gravenstein, N.; Seubert, C.N.; Morey, T.E.; Sumners, C.; Vasilopoulos, T.; Yang, J.-J.; Martynyuk, A.E. Role of environmental stressors in determining the developmental outcome of neonatal anesthesia. *Psychoneuroendocrinology* **2017**, *81*, 96–104. https://doi.org/10.1016/j.psyneuen.2017.04.001.
10. Yang, J.; Ju, L.; Jia, M.; Zhang, H.; Sun, X.; Ji, M.; Yang, J.; Martynyuk, A.E. Subsequent maternal separation exacerbates neurobehavioral abnormalities in rats neonatally exposed to sevoflurane anesthesia. *Neurosci. Lett.* **2017**, *661*, 137–142, https://doi.org/10.1016/j.neulet.2017.09.063.
11. Yang, J.; Ju, L.; Yang, C.; Xue, J.; Setlow, B.; Morey, T.E.; Gravenstein, N.; Seubert, C.N.; Vasilopoulos, T.; Martynyuk, A.E. Effects of combined brief etomidate anesthesia and postnatal stress on amygdala expression of Cl− cotransporters and corticotropin-releasing hormone and alcohol intake in adult rats. Neurosci. Lett. **2018**, *685*, 83–89. https://doi.org/10.1016/j.neulet.2018.08.019.
12. Xu, N.; Lei, L.; Lin, Y.; Ju, L.-S.; Morey, T.E.; Gravenstein, N.; Yang, J.; Martynyuk, A.E. A Methyltransferase Inhibitor (Decitabine) Alleviates Intergenerational Effects of Paternal Neonatal Exposure to Anesthesia With Sevoflurane. *Obstet. Anesthesia Dig.* **2020**, *131*, 1291–1299. https://doi.org/10.1213/ane.0000000000005097.
